# Progression of Behavioral Disturbances and Neuropsychiatric Symptoms in Patients With Genetic Frontotemporal Dementia

**DOI:** 10.1001/jamanetworkopen.2020.30194

**Published:** 2021-01-06

**Authors:** Alberto Benussi, Enrico Premi, Stefano Gazzina, Chiara Brattini, Elisa Bonomi, Antonella Alberici, Lize Jiskoot, John C. van Swieten, Raquel Sanchez-Valle, Fermin Moreno, Robert Laforce, Caroline Graff, Matthis Synofzik, Daniela Galimberti, Mario Masellis, Carmela Tartaglia, James B. Rowe, Elizabeth Finger, Rik Vandenberghe, Alexandre de Mendonça, Fabrizio Tagliavini, Isabel Santana, Simon Ducharme, Chris R. Butler, Alexander Gerhard, Johannes Levin, Adrian Danek, Markus Otto, Giovanni Frisoni, Roberta Ghidoni, Sandro Sorbi, Isabelle Le Ber, Florence Pasquier, Georgia Peakman, Emily Todd, Martina Bocchetta, Jonathan D. Rohrer, Barbara Borroni

**Affiliations:** 1 Centre for Neurodegenerative Disorders, Department of Clinical and Experimental Sciences, University of Brescia, Brescia, Italy; 2 Vascular Neurology Unit, Department of Neurological and Vision Sciences, ASST Spedali Civili, Brescia, Italy; 3 Neurophysiology Unit, Department of Neurological and Vision Sciences, ASST Spedali Civili, Brescia, Italy; 4 Neurology Unit, Department of Neurological and Vision Sciences, ASST Spedali Civili, Brescia, Italy; 5 Department of Neurology, Erasmus Medical Centre, Rotterdam, the Netherlands; 6 Alzheimer’s Disease and Other Cognitive Disorders Unit, Neurology Service, Hospital Clínic, Institut d’Investigacións Biomèdiques August Pi I Sunyer, University of Barcelona, Barcelona, Spain; 7 Cognitive Disorders Unit, Department of Neurology, Donostia University Hospital, San Sebastian, Spain; 8 Neuroscience Area, Biodonostia Health Research Institute, San Sebastian, Gipuzkoa, Spain; 9 Clinique Interdisciplinaire de Mémoire, Département des Sciences Neurologiques du CHU de Québec, and Faculté de Médecine, Université Laval, Québec, Canada; 10 Center for Alzheimer Research, Division of Neurogeriatrics, Department of Neurobiology, Care Sciences and Society, Bioclinicum, Karolinska Institutet, Solna, Sweden; 11 Unit for Hereditary Dementias, Theme Aging, Karolinska University Hospital, Solna, Sweden; 12 Department of Neurodegenerative Diseases, Hertie-Institute for Clinical Brain Research and Center of Neurology, University of Tübingen, Tübingen, Germany; 13 Center for Neurodegenerative Diseases, Tübingen, Germany; 14 Fondazione Ca’ Granda, IRCCS Ospedale Policlinico, Milan, Italy; 15 University of Milan, Centro Dino Ferrari, Milan, Italy; 16 Sunnybrook Health Sciences Centre, Sunnybrook Research Institute, University of Toronto, Toronto, Ontario, Canada; 17 Tanz Centre for Research in Neurodegenerative Diseases, University of Toronto, Toronto, Ontario, Canada; 18 Department of Clinical Neurosciences, University of Cambridge, United Kingdom; 19 Department of Clinical Neurological Sciences, University of Western Ontario, London, Canada; 20 Laboratory for Cognitive Neurology, Department of Neurosciences, KU Leuven, Leuven, Belgium; 21 Neurology Service, University Hospitals Leuven, Leuven, Belgium; 22 Leuven Brain Institute, KU Leuven, Leuven, Belgium; 23 Faculty of Medicine, University of Lisbon, Lisbon, Portugal; 24 Fondazione IRCCS Istituto Neurologico Carlo Besta, Milano, Italy; 25 Neurology Service, Faculty of Medicine, University Hospital of Coimbra, University of Coimbra, Coimbra, Portugal; 26 Center for Neuroscience and Cell Biology, Faculty of Medicine, University of Coimbra, Coimbra, Portugal; 27 Department of Psychiatry, McGill University Health Centre, McGill University, Montreal, Québec, Canada; 28 McConnell Brain Imaging Centre, Montreal Neurological Institute, McGill University, Montreal, Québec, Canada; 29 Nuffield Department of Clinical Neurosciences, Medical Sciences Division, University of Oxford, Oxford, United Kingdom; 30 Department of Brain Sciences, Imperial College London, London, United Kingdom; 31 Division of Neuroscience and Experimental Psychology, Wolfson Molecular Imaging Centre, University of Manchester, Manchester, United Kingdom; 32 Departments of Geriatric Medicine and Nuclear Medicine, University of Duisburg-Essen, Duisburg, Germany; 33 Department of Neurology, Ludwig-Maximilians Universität München, Munich, Germany; 34 German Center for Neurodegenerative Diseases, Munich, Germany; 35 Munich Cluster of Systems Neurology, Munich, Germany; 36 Department of Neurology, University of Ulm, Ulm, Germany; 37 IRCCS Centro San Giovanni di Dio Fatebenefratelli, Brescia, Italy; 38 Molecular Markers Laboratory, IRCCS Istituto Centro San Giovanni di Dio Fatebenefratelli, Brescia, Italy; 39 Department of Neurofarba, University of Florence, Florence, Italy; 40 IRCCS Fondazione Don Carlo Gnocchi, Florence, Italy; 41 Institut National de la Santé et de la Recherche Medicale (INSERM) U1127, Paris, France; 42 Centre de National de la Recherche Scientifique, Unité Mixte de Recherche (UMR) 7225, Paris, France; 43 Unité Mixte de Recherche en Santé 1127, Université Pierre et Marie Curie (Paris 06), Sorbonne Universités, Paris, France; 44 Institute du Cerveau et de la Moelle Epinière, Paris, France; 45 Inserm CHU Lille, Lille Neurosciences & Cognition UMR-S1172 Degenerative and Vascular Cognitive Disorders, Université de Lille, Lille, France; 46 CHU Lille, DistAlz Licend Memory Clinic, Lille, France; 47 Department of Neurodegenerative Disease, Dementia Research Centre, UCL Institute of Neurology, Queen Square, London, United Kingdom

## Abstract

**Importance:**

Behavioral disturbances are core features of frontotemporal dementia (FTD); however, symptom progression across the course of disease is not well characterized in genetic FTD.

**Objective:**

To investigate behavioral symptom frequency and severity and their evolution and progression in different forms of genetic FTD.

**Design, Setting, and Participants:**

This longitudinal cohort study, the international Genetic FTD Initiative (GENFI), was conducted from January 30, 2012, to May 31, 2019, at 23 multicenter specialist tertiary FTD research clinics in the United Kingdom, the Netherlands, Belgium, France, Spain, Portugal, Italy, Germany, Sweden, Finland, and Canada. Participants included a consecutive sample of 232 symptomatic FTD gene variation carriers comprising 115 with variations in *C9orf72*, 78 in *GRN*, and 39 in *MAPT*. A total of 101 carriers had at least 1 follow-up evaluation (for a total of 400 assessments). Gene variations were included only if considered pathogenetic.

**Main Outcomes and Measures:**

Behavioral and neuropsychiatric symptoms were assessed across disease duration and evaluated from symptom onset. Hierarchical generalized linear mixed models were used to model behavioral and neuropsychiatric measures as a function of disease duration and variation.

**Results:**

Of 232 patients with FTD, 115 (49.6%) had a *C9orf72* expansion (median [interquartile range (IQR)] age at evaluation, 64.3 [57.5-69.7] years; 72 men [62.6%]; 115 White patients [100%]), 78 (33.6%) had a *GRN* variant (median [IQR] age, 63.4 [58.3-68.8] years; 40 women [51.3%]; 77 White patients [98.7%]), and 39 (16.8%) had a *MAPT* variant (median [IQR] age, 56.3 [49.9-62.4] years; 25 men [64.1%]; 37 White patients [94.9%]). All core behavioral symptoms, including disinhibition, apathy, loss of empathy, perseverative behavior, and hyperorality, were highly expressed in all gene variant carriers (>50% patients), with apathy being one of the most common and severe symptoms throughout the disease course (51.7%-100% of patients). Patients with *MAPT* variants showed the highest frequency and severity of most behavioral symptoms, particularly disinhibition (79.3%-100% of patients) and compulsive behavior (64.3%-100% of patients), compared with *C9orf72* carriers (51.7%-95.8% of patients with disinhibition and 34.5%-75.0% with compulsive behavior) and *GRN* carriers (38.2%-100% with disinhibition and 20.6%-100% with compulsive behavior). Alongside behavioral symptoms, neuropsychiatric symptoms were very frequently reported in patients with genetic FTD: anxiety and depression were most common in *GRN* carriers (23.8%-100% of patients) and *MAPT* carriers (26.1%-77.8% of patients); hallucinations, particularly auditory and visual, were most common in *C9orf72* carriers (10.3%-54.5% of patients). Most behavioral and neuropsychiatric symptoms increased in the early-intermediate phases and plateaued in the late stages of disease, except for depression, which steadily declined in *C9orf72* carriers, and depression and anxiety, which surged only in the late stages in *GRN* carriers.

**Conclusions and Relevance:**

This cohort study suggests that behavioral and neuropsychiatric disturbances differ between the common FTD gene variants and have different trajectories throughout the course of disease. These findings have crucial implications for counseling patients and caregivers and for the design of disease-modifying treatment trials in genetic FTD.

## Key Points

**Question:**

Do behavioral and neuropsychiatric symptoms evolve differently in patients with distinct genetic variations for frontotemporal dementia?

**Findings:**

In this cohort study of 232 patients with genetic frontotemporal dementia, patients with *MAPT* variants showed the highest frequency and severity of most behavioral symptoms compared with *C9orf72* and *GRN* carriers. Anxiety and depression were most common in *GRN* and *MAPT* carriers; hallucinations, particularly auditory and visual, were most common in *C9orf72* carriers.

**Meaning:**

These findings suggest that behavioral and neuropsychiatric disturbances differ between the common frontotemporal dementia gene variations and have different trajectories through the course of disease.

## Teaser

This cohort study uses data from tertiary frontotemporal dementia research clinics across Europe and Canada to assess the frequency and severity of behavioral symptoms and their progression in different forms of genetic frontotemporal dementia.

## Introduction

Frontotemporal dementia (FTD) encompasses a heterogeneous group of neurodegenerative disorders with a wide range of clinical, genetic, and neuropathological features.^1^ Approximately one-third of patients with FTD have an autosomal dominant family history,^2^ with variations of 3 main genes, microtubule-associated protein tau (*MAPT*), granulin (*GRN*), and chromosome 9 open reading frame 72 (*C9orf72*), together accounting for 10% to 20% of all FTD and 70% of all genetic FTD cases.^3,4^ Behavioral and personality changes are among the most prominent symptoms in FTD, particularly in the behavioral variant FTD,^5^ but are also seen in the primary progressive aphasias,^6^ in which behavioral symptoms are frequently associated with speech and language deficits.^7,8,9,10^

Irrespective of the particular presenting syndrome, these disturbances progress over time, with symptoms changing over the course of the disease.^11,12,13^ Several studies have investigated the development of behavioral disturbances in sporadic FTD,^14,15,16,17,18,19^ with a recent study showing that the progression and severity of behavioral symptoms may change during the course of the disease.^20^ Negative symptoms, such as apathy and loss of empathy, steadily increase throughout the course of disease, whereas positive symptoms, such as disinhibition and perseverative behavior, tend to worsen until the intermediate stages and then decrease in severity in the more advanced phases.^20^

However, this progression has not been systematically addressed within and across the symptomatic phases of genetic FTD, and it is currently unclear how behavioral and neuropsychiatric symptoms change during the course of the disease and whether different gene variants have distinct patterns of symptom progression. This question has crucial implications for counseling patients and caregivers and should be pivotal when designing clinical outcomes and monitoring measures for disease-modifying treatment trials for each specific gene. The aim of the present study was to investigate and characterize the frequency, evolution, and progression of behavioral and neuropsychiatric symptoms in a large cohort of patients with genetic FTD in the international Genetic FTD Initiative (GENFI),^21^ hypothesizing that the nature and severity of behavioral disturbances may follow different trajectories depending on the gene variant.

## Methods

### Participants

In this longitudinal cohort study, patients were recruited from 23 multicenter specialist tertiary FTD research clinics in the United Kingdom, the Netherlands, Belgium, France, Spain, Portugal, Italy, Germany, Sweden, Finland, and Canada. From the GENFI study^21^ data freeze 5 (from January 30, 2012, to May 31, 2019), a consecutive sample of 232 symptomatic participants were included, comprising 115 with gene variations in *C9orf72*, 78 in *GRN*, and 39 in *MAPT*. Gene variants were included only if considered pathogenetic (full inclusion and exclusion criteria are reported in the eMethods and eTables 1 and 2 in the Supplement).

Patients were considered symptomatic when the assessing clinician felt that the patient had evidence of progressive cognitive or behavioral change. All participants underwent genetic testing to determine whether they were a carrier or noncarrier. All participants underwent the GENFI standardized assessment.^21^ During the first visit, demographic information of all participants was collected, as well as information regarding clinical background (neuropsychiatric features, family and medical history, medication, and onset symptoms).

Local ethics committees approved the study at each site, and all participants provided written informed consent. The study was conducted according to the Declaration of Helsinki^22^ and followed the Strengthening the Reporting of Observational Studies in Epidemiology (STROBE) reporting guideline.

### Clinical Evaluation

Participants underwent a clinical and cognitive assessment to evaluate their symptomatic status and cognitive performance at baseline and then at follow-up (232 with baseline assessment, 101 with at least 2 evaluations, 35 with at least 3 evaluations, 15 with at least 4 evaluations, 8 with at least 5 evaluations, 7 with at least 6 evaluations, and 3 with 7 evaluations), for a total of 400 evaluations (eTable 3 in the Supplement).

In all patients, both behavioral and neuropsychiatric symptoms were assessed, and severity was rated on a 5-point scale (0 = absent, 0.5 = questionable or very mild, 1 = mild, 2 = moderate, and 3 = severe). Behavioral symptoms included disinhibition, apathy, loss of sympathy or empathy, compulsive or ritualistic behavior, hyperorality, and dietary changes. Neuropsychiatric symptoms included visual, auditory, and tactile hallucinations; delusions; depression; and anxiety. We measured functional status using the Frontotemporal Dementia Rating Scale, which has a very high interrater variability (intraclass correlation coefficient of 0.994).^17^

### Statistical Analysis

Baseline demographic and clinical variables were compared across groups using the Kruskal-Wallis *H* test or Fisher test, as appropriate.

Hierarchical generalized linear mixed models were used to model behavioral and neuropsychiatric measures as a function of disease duration, evaluated in years from symptom onset, and gene variation (*C9orf72*, *GRN*, or *MAPT*). A gamma regression was applied owing to the skewed distribution of behavioral and neuropsychiatric measures.^23^ As previously reported, possible nonlinear changes, such as quadratic and cubic relations, could be expected over time for each measure.^20,21^ Accordingly, possible 2-factor and 3-factor interaction terms along with second- and third-order terms were examined to reach a final model that fit the data well (eTable 4 in the Supplement). A penalized likelihood method (bayesian information criteron) was also considered to evaluate the model fit.^24^ Considering that some participants were recruited from the same family, the family membership was included in the model as a random effect, as it was expected that members from the same family might have covariance in symptom intensity and progression due to a shared genetic and environmental background. A random-effects model was used to estimate the variance in the effect of a variable between different clusters in the data, and this estimation allowed for correlation in the outcome between members of the same cluster.^25,26^

We performed Satterthwaite approximations with robust covariances for each model to assess whether the mean value of the measure differed between gene variants. We predicted average values from the mixed-effects model for each group and differences between gene variants at different time points (0-4 years, 4-8 years, and 8-12 years of disease duration, evaluated from symptom onset). We applied Bonferroni adjustments for multiple comparisons, with an adjusted α level of .0028 (18 comparisons per symptom). All time points were arbitrarily defined in order to distribute an equal number of patients in the 3 categories, roughly corresponding to an early, intermediate, and late phase.

Statistical significance was assumed at *P* < .05, and *P* values were 2-sided. Data analyses were carried out using SPSS, version 25.0 (IBM Corp) and GraphPad Prism, version 8.0 (GraphPad Software).

## Results

Of 232 patients with FTD, 115 (49.6%) had a *C9orf72* expansion (median [interquartile range (IQR)] age at evaluation, 64.3 [57.5-69.7] years; 72 men [62.6%]; 115 White patients [100%]), 78 (33.6%) had a *GRN* variant (median [IQR] age, 63.4 [58.3-68.8] years; 40 women [51.3%]; 77 White patients [98.7%]), and 39 (16.8%) had a *MAPT* variant (median [IQR] age, 56.3 [49.9-62.4] years; 25 men [64.1%]; 37 White patients [94.9%]). Demographic characteristics of the patients included are reported in Table 1. There were significant differences in age at symptom onset between groups, with *GRN* (median [IQR] age, 60.0 [55.0-66.0] years; *P* < .001) and *C9orf72* carriers (median [IQR] age, 59.0 [53.0-65.0] years) being significantly older than *MAPT* carriers (median [IQR] age, 52.0 [45.0-56.0] years). Patients with variations in *MAPT* (median [IQR] duration, 3.3 [1.7-7.6] years) and *C9orf72* (median [IQR] duration, 3.9 [2.2-5.9] years) expansions had a longer disease duration at baseline compared with *GRN* carriers (median [IQR] duration, 2.4 [1.4-3.5] years; *P* = .001). No differences were observed between groups in terms of sex, years of education, or disease severity evaluated with the Frontotemporal Dementia Rating Scale. Disease severity was not significantly different between sexes, both in the whole cohort of patients and within specific genetic groups.

**Table 1.** Baseline Demographic and Clinical Characteristics of Patients With FTD

Characteristic	Median (IQR)^a^		
	*C9orf72* (n = 115)	*GRN* (n = 78)	*MAPT* (n = 39)
Female sex, No. (%)	43 (37.4)	40 (51.3)	14 (35.9)
Ethnicity, No. (%)			
White	115 (100)	77 (98.7)	37 (94.9)
African	0	0	2 (5.1)
Indian	0	1 (1.3)	0
Education, y	13.0 (11.0-15.0)	12.0 (8.0-15.0)	13.0 (11.0-16.0)
Age at symptom onset, y	59.0 (53.0-65.0)^b^	60.0 (55.0-66.0)	52.0 (45.0-56.0)^c^^,^^d^
Disease duration, y	3.9 (2.2-5.9)^c^	2.4 (1.4-3.5)^b^^,^^d^	3.3 (1.7-7.6)^d^
Person-years of follow-up	93.8	47.0	47.9
Frontotemporal Dementia Rating Scale, %	38.0 (17.0-60.0)	46.5 (22.8-68.1)	40.0 (25.0-57.0)

Abbreviations: *C9orf72*, chromosome 9 open reading frame 72; FTD, frontotemporal dementia; *GRN*, granulin; IQR, interquartile range; *MAPT*, microtubule-associated protein tau.

^a^ Values are listed as median (IQR) unless otherwise specified.

^b^ *P* < .05 vs *MAPT* pairwise comparisons after significant interaction at the Kruskal-Wallis *H* test or at the Fisher exact test, after adjustment for multiple comparisons.

^c^ *P* < .05 vs *GRN*.

^d^ *P* < .05 vs *C9orf72*.

### Frequency of Behavioral and Neuropsychiatric Symptoms

**Figure 1.**
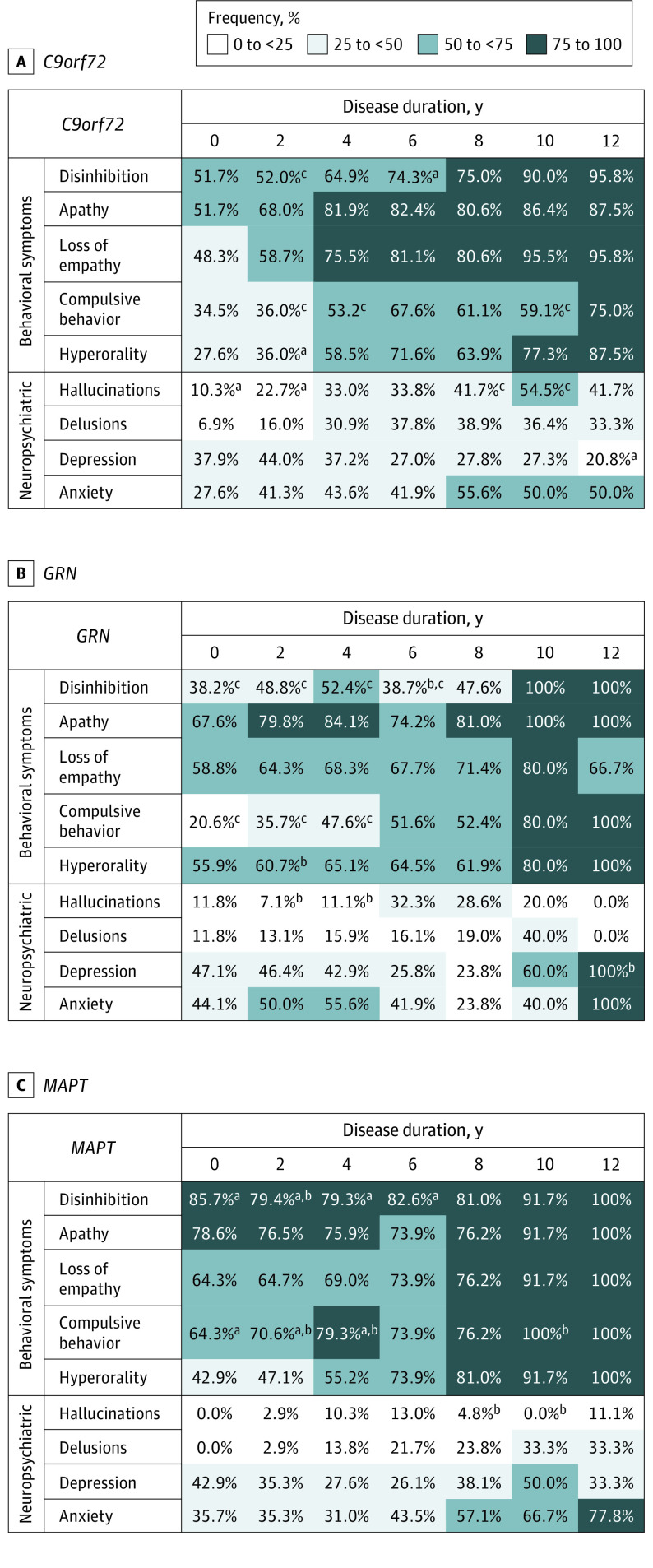
Frequency of Behavioral and Neuropsychiatric Symptoms in A, *C9orf72* Expansion; B, *GRN*; and C, *MAPT* Carriers *C9orf72* indicates chromosome 9 open reading frame 72; *GRN*, granulin; *MAPT*, microtubule-associated protein tau. ^a^*P* < .05 vs *GRN.* ^b^*P* < .05 vs *C9orf72.* ^c^*P* < .05 vs *MAPT* pairwise comparisons after significant interaction at the Fisher exact test, after adjustment for multiple comparisons.

The frequencies of behavioral and neuropsychiatric symptoms are reported in Figure 1 for each gene variant, expressed as percentage of patients reporting that particular symptom out of the total number of patients with that specific disease duration, evaluated from symptom onset.

#### C9orf72

We observed a high frequency (27.6%-95.8% of patients) of all behavioral symptoms in *C9orf72* carriers, particularly in the intermediate and late stages of disease (74.3%-95.8% of patients with disinhibition, 82.4%-87.5% with apathy, 81.1%-95.8% with loss of empathy, 67.6%-75.0% with compulsive behavior, 71.6%-87.5% with hyperorality), with only slightly lower frequencies for compulsive behavior and hyperorality in the first 4 years of the disease (34.5%-53.2% of patients with compulsive behavior, 27.6%-58.5% with hyperorality). Depression and anxiety were present in approximately one-third of patients in the early stages (37.2%-44.0% of patients with depression, 27.6%-43.6% with anxiety), whereas in the intermediate stages, all neuropsychiatric symptoms were evenly distributed (33.8%-41.7% of patients with hallucinations, 37.8%-38.9% with delusions, 27.0%-27.8% with depression, 41.9%-55.6% with anxiety). By the late stages, anxiety and hallucinations predominated (50.0% of patients with anxiety, 41.7%-54.5% with hallucinations) (Figure 1A).

#### GRN

Apathy, loss of empathy, and hyperorality were the most frequent symptoms, already present in more than 50% of patients during the early stages (67.6%-84.1% of patients with apathy, 58.8%-68.3% with loss of empathy, 55.9%-65.1% with hyperorality). In the late stages, all behavioral symptoms were seen in nearly all patients (100% of patients with disinhibition, 100% with apathy, 66.7%-80.0% with loss of empathy, 80.0%-100% with compulsive behavior, 80.0%-100% with hyperorality). Depression and anxiety were the most frequent neuropsychiatric symptoms, particularly in the early (42.9%-47.1% of patients with depression, 44.1%-55.6% with anxiety) and late (60.0%-100% of patients with depression, 40.0%-100% with anxiety) phases, when compared with hallucinations (0.0%-32.3% of patients) and delusions (0.0%-40.0% of patients) (Figure 1B).

#### MAPT

The frequency of behavioral disturbances was extremely high in patients with *MAPT* gene variations, with more than 60% of patients reporting all symptoms in the early disease stages (79.3%-85.7% of patients with disinhibition, 75.9%-78.6% with apathy, 64.3%-69.0% with loss of empathy, 64.3%-79.3% with compulsive behavior), except for hyperorality (42.9%-55.2% of patients). By contrast, hallucinations (0.0%-13.0% of patients) and delusions (0.0%-33.3% of patients) were not at all frequent during the course of the disease. Depression and anxiety were moderately represented during the entire course of the disease (26.1%-50.0% of patients with depression, 31.0%-77.8% with anxiety), with the latter increasing in the final stages (66.7%-77.8% of patients) (Figure 1C).

### Longitudinal Behavioral Changes

**Figure 2.**
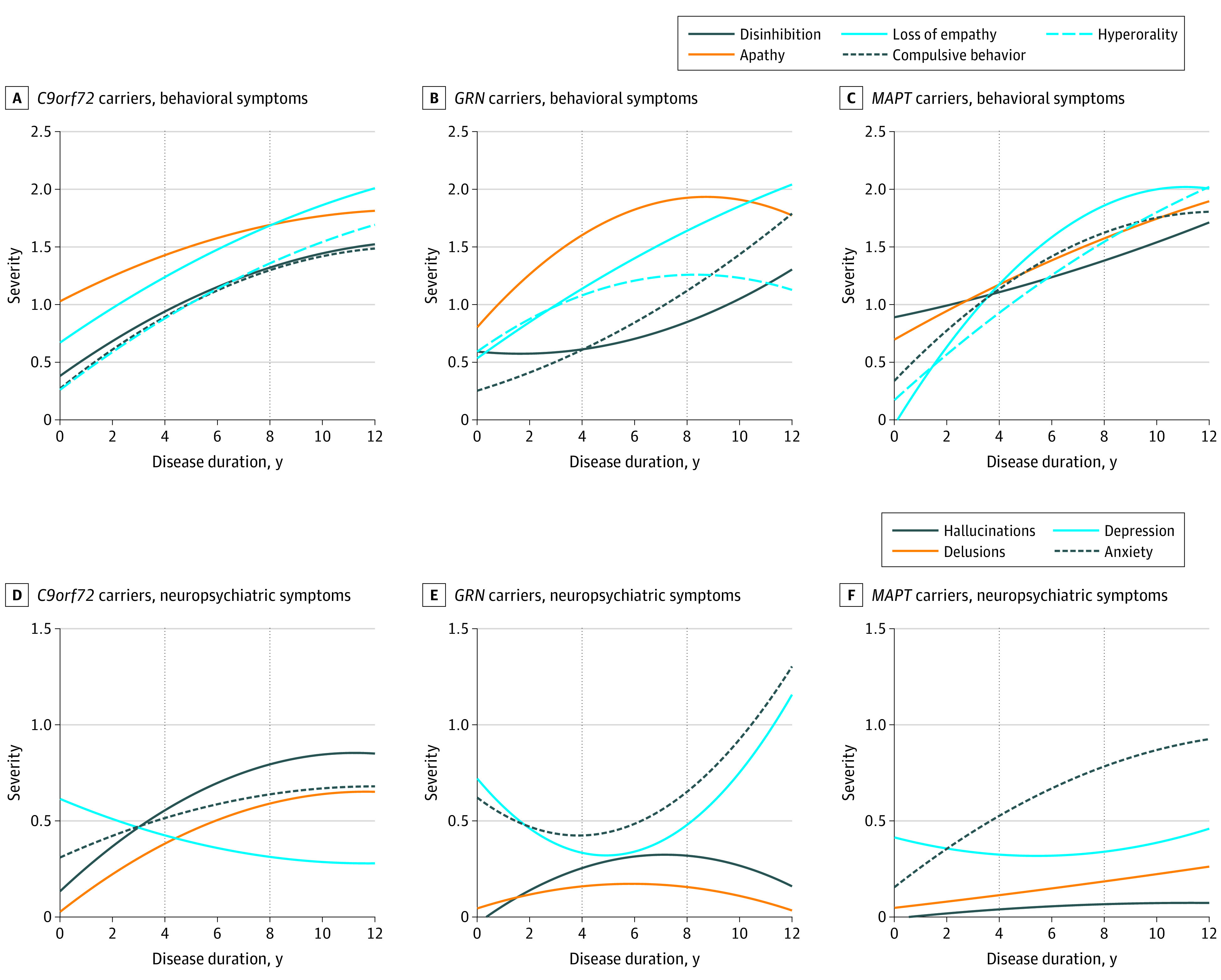
Predicted Behavioral (A, *C9orf72* carriers; B, *GRN* carriers; C, *MAPT* carriers) and Neuropsychiatric Symptom (D, *C9orf72* carriers; E, *GRN* carriers; F, *MAPT* carriers) Severity According to Disease Duration *C9orf72* indicates chromosome 9 open reading frame 72; *GRN*, granulin; *MAPT*, microtubule-associated protein tau.

Estimates of longitudinal changes in behavioral symptoms are reported in Figure 2A, 2B, and 2C for each gene variant, expressed as average severity for each symptom on a 5-point scale (0, 0.5, 1, 2, and 3) as a function of disease duration and evaluated from symptom onset.

#### C9orf72

In the early phases of disease (0-4 years), we observed a significant predominance of apathy (mean severity score: 1.22 [95% CI, 1.05-1.38] points) compared with other behavioral symptoms in *C9orf72* carriers, which tended to plateau in the intermediate (mean severity score at 4-8 years: 1.67 [95% CI, 1.52-1.82] points) and late (mean severity score at 8-12 years: 1.62 [95% CI, 1.47-1.78] points) stages, when loss of empathy or sympathy became the predominant behavioral symptom (mean severity score at 4-8 years: 1.58 [95% CI, 1.45-1.70] points; at 8-12 years: 1.92 [95% CI, 1.79-2.04] points) (Table 2 and Figure 2A). All other behavioral symptoms, such as disinhibition, compulsive behavior, and hyperorality, had similar trajectories, being less severe and steadily increasing in the early phases and then slowing down in the intermediate-late phases.

**Table 2.** Estimates of Behavioral and Neuropsychiatric Symptoms’ Severity in Patients With FTD

Symptom	Mean (95% CI) severity score		
	0-4 y From symptom onset	4-8 y From symptom onset	8-12 y From symptom onset
***C9orf72***			
Disinhibition	0.67 (0.54-0.80)^a^^,^^b^^,^^c^	1.20 (1.05-1.35)^b^^,^^d^^,^^e^	1.37 (1.22-1.52)^a^^,^^d^
Apathy	1.22 (1.05-1.38)^a^^,^^b^	1.67 (1.52-1.82)^d^	1.62 (1.47-1.78)^d^
Loss of empathy	0.94 (0.81-1.07)^a^^,^^b^	1.58 (1.45-1.70)^b^^,^^d^	1.92 (1.79-2.04)^a^^,^^d^
Compulsive behavior	0.62 (0.49-0.74)^a^^,^^b^	1.16 (1.01-1.30)^d^	1.28 (1.09-1.46)^d^
Hyperorality	0.54 (0.43-0.65)^a^^,^^b^^,^^e^	1.26 (1.11-1.40)^d^	1.57 (1.35-1.79)^d^
Hallucinations	0.41 (0.25-0.57)^e^	0.61 (0.41-0.82)	0.75 (0.41-1.10)^c^
Delusions	0.22 (0.14-0.29)^a^	0.52 (0.39-0.66)^c^^,^^d^^,^^e^	0.50 (0.32-0.68)
Depression	0.53 (0.45-0.61)^a^	0.32 (0.25-0.38)^d^	0.38 (0.27-0.49)
Anxiety	0.47 (0.39-0.56)^b^	0.51 (0.45-0.57)^b^	0.65 (0.51-0.80)^a^^,^^d^
***GRN***			
Disinhibition	0.61 (0.49-0.74)^c^	0.62 (0.40-0.83)	1.07 (0.59-1.55)
Apathy	1.34 (1.19-1.49)^c^	1.69 (1.37-2.01)	1.93 (1.57-2.29)
Loss of empathy	0.93 (0.82-1.04)^a^	1.29 (1.02-1.55)^d^	1.81 (1.23-2.39)^a^^,^^d^
Compulsive behavior	0.48 (0.38-0.58)^a^^,^^c^	0.75 (0.57-0.93)^d^	1.43 (0.67-2.19)
Hyperorality	0.95 (0.82-1.09)^f^	1.01 (0.79-1.22)	1.36 (0.87-1.85)
Hallucinations	0.14 (0.06-0.21)^a^^,^^f^	0.37 (0.20-0.55)^c^^,^^d^	0.18 (0.00-0.40)
Delusions	0.12 (0.07-0.17)^c^^,^^f^	0.17 (0.05-0.29)^f^	0.11 (0.00-0.23)
Depression	0.48 (0.42-0.55)	0.27 (0.17-0.37)	0.78 (0.11-1.46)
Anxiety	0.51 (0.43-0.58)	0.39 (0.29-0.50)	0.95 (0.05-1.84)
***MAPT***			
Disinhibition	1.01 (0.83-1.19)^e^^,^^f^	1.26 (1.00-1.52)^e^	1.86 (1.58-2.15)^e^^,^^f^
Apathy	0.92 (0.75-1.09)^b^^,^^e^	1.35 (1.04-1.66)^b^	2.07 (1.93-2.22)^a^^,^^d^
Loss of empathy	0.71 (0.55-0.88)^a^^,^^b^	1.37 (0.97-1.76)^d^	1.82 (1.37-2.27)^d^
Compulsive behavior	0.80 (0.67-0.93)^b^^,^^e^	1.39 (1.09-1.69)^b^	1.69 (1.44-1.94)^a^^,^^d^
Hyperorality	0.55 (0.42-0.68)^a^^,^^b^	1.36 (1.07-1.65)^b^^,^^d^	2.09 (1.87-2.30)^a^^,^^d^
Hallucinations	0.02 (0.00-0.04)^e^^,^^f^	0.06 (0.00-0.12)^e^	0.06 (0.00-0.13)^f^
Delusions	0.06 (0.00-0.14)	0.25 (0.08-0.43)^f^	0.23 (0.14-0.32)
Depression	0.34 (0.24-0.44)	0.31 (0.17-0.46)^b^	0.61 (0.47-0.76)^a^
Anxiety	0.38 (0.28-0.48)^b^	0.58 (0.42-0.74)^b^	0.97 (0.88-1.05)^a^^,^^d^

Abbreviations: *C9orf72*, chromosome 9 open reading frame 72; FTD, frontotemporal dementia; *GRN*, granulin; *MAPT*, microtubule-associated protein tau.

^a^ *P* < .05 vs 4-8 years.

^b^ *P* < .05 vs 8-12 years.

^c^ *P* < .05 vs *MAPT*.

^d^ *P* < .05 vs 0-4 years.

^e^ *P* < .05 vs *GRN*.

^f^ *P* < .05 vs *C9orf72*; pairwise contrasts after adjustment for multiple comparisons.

#### GRN

Similar to *C9orf72, GRN* carriers had apathy as the predominant symptom (mean severity score at 1-4 years: 1.34 [95% CI, 1.19-1.49] points), which increased into intermediate-late stages (mean severity score at 4-8 years: 1.69 [95% CI, 1.37-2.01] points; at 8-12 years: 1.93 [95% CI, 1.57-2.29] points). Loss of empathy (mean severity score: 0.93 [95% CI, 0.82-1.04] points) and hyperorality (mean severity score: 0.95 [95% CI, 0.82-1.09] points) were the next most severe behavioral symptoms in the early disease stages, with loss of empathy steadily increasing over the course of the disease (mean severity score at 4-8 years: 1.29 [95% CI, 1.02-1.55] points; at 8-12 years: 1.81 [95% CI, 1.23-2.39] points), whereas hyperorality remained stable (mean severity score at 4-8 years: 1.01 [95% CI, 0.79-1.22] points; at 8-12 years: 1.36 [95% CI, 0.87-1.85] points). Compulsive behavior and disinhibition were less pronounced in the early and intermediate stages, and increased in the late phases (mean severity score at 0-4 years: compulsive behavior, 0.48 [95% CI, 0.38-0.58] points; disinhibition, 0.61 [95% CI, 0.49-0.74] points; at 4-8 years: compulsive behavior, 0.75 [95% CI, 0.57-0.93] points; disinhibition, 0.62 [95% CI, 0.40-0.83] points; at 8-12 years: compulsive behavior, 1.43 [95% CI, 0.67-2.19] points; disinhibition, 1.07 [95% CI, 0.59-1.55] points) (Table 2 and Figure 2B).

#### MAPT

In *MAPT* carriers, disinhibition was the predominant symptom in the early phase (mean severity score: 1.01 [95% CI, 0.83-1.19] points), as compared with *C9orf72* (mean severity score: 0.67 [95% CI, 0.54-0.80] points) and *GRN* carriers (mean severity score: 0.61 [95% CI, 0.49-0.74] points). Compulsive behavior was also significantly increased in the early phase (mean severity score: 0.80 [95% CI, 0.67-0.93] points) compared with the other 2 variations (mean severity score: C9orf72, 0.62 [95% CI, 0.49-0.74] points; GRN, 0.48 [95% CI, 0.38-0.58] points). In the intermediate and late phases, all behavioral symptoms progressively worsened following similar trajectories, and hyperorality was significantly increased in the late phase (mean severity score: 2.09 [95% CI, 1.87-2.30] points) compared with *C9orf72* (mean severity score: 1.57 [95% CI, 1.35-1.79] points) and *GRN* (mean severity score: 1.36 [95% CI, 0.87-1.85] points) carriers (Table 2 and Figure 2C).

### Longitudinal Changes in Neuropsychiatric Symptoms

Estimates of longitudinal changes in neuropsychiatric symptoms are reported in Figure 2D, 2E, 2F, and Table 2 for each variation, expressed as mean severity for each symptom on a 5-point scale (0, 0.5, 1, 2, and 3) as a function of disease duration, evaluated from symptom onset (significant pairwise comparisons are reported in eFigure 1 in the Supplement as Circos plots.^27^)

#### C9orf72

In the early phase of disease, depression was the predominant symptom in *C9orf72* and tended to steadily decline in the intermediate and late phases (mean severity score at 0-4 years: 0.53 [95% CI, 0.45-0.61] points; at 4-8 years: 0.32 [95% CI, 0.25-0.38] points; at 8-12 years: 0.38 [95% CI, 0.27-0.49] points), when hallucinations tended to prevail over other neuropsychiatric symptoms (mean severity score at 4-8 years: 0.61 [95% CI, 0.41-0.82] points; at 8-12 years: 0.75 [95% CI, 0.41-1.10] points) (Table 2 and Figure 2D). All other symptoms tended to plateau in the late stages of disease, being less severe than hallucinations. Auditory and visual hallucinations steadily increased during the course of the disease (mean severity score at 0-4 years: auditory hallucinations, 0.22 [95% CI, 0.14-0.31] points; visual hallucinations, 0.21 [95% CI, 0.14-0.27] points; at 4-8 years: auditory hallucinations, 0.23 [95% CI, 0.14-0.31] points; visual hallucinations, 0.24 [95% CI, 0.17-0.30] points; at 8-12 years: auditory hallucinations, 0.23 [95% CI, 0.14-0.31] points; visual hallucinations, 0.31 [95% CI, 0.22-0.39] points), whereas tactile hallucinations were not frequently reported (mean severity score at 0-4 years: 0.04 [95% CI, 0.01-0.2] points; at 4-8 years: 0.03 [95% CI, 0.01-0.15] points; at 8-12 years: 0.02 [95% CI, 0.01-0.18] points) (eFigure 2A in the Supplement).

#### GRN

Anxiety and depression significantly increased in the early stages of disease, gradually decreasing in the intermediate stages and subsequently increasing again in the late stages (mean severity score at 0-4 years: anxiety, 0.51 [95% CI, 0.43-0.58] points; depression, 0.48 [95% CI, 0.42-0.55] points; at 4-8 years: anxiety, 0.39 [95% CI, 0.29-0.50] points; depression, 0.27 [95% CI, 0.17-0.37] points; at 8-12 years: anxiety, 0.95 [95% CI, 0.05-1.84] points; depression, 0.78 [95% CI, 0.11-1.46] points). Delusions and, to a lesser extent, hallucinations were less prominent when compared with *C9orf72* carriers, particularly in the early and late stages (mean severity score for delusions at 0-4 years: 0.12 [95% CI, 0.07-0.17] points in *GRN* carriers vs 0.22 [95% CI, 0.14-0.29] points in *C9orf72*; at 8-12 years: 0.11 [95% CI, 0.00-0.23] points in *GRN* carriers vs 0.50 [95% CI, 0.32-0.68] points in *C9orf72* carriers) (Table 2 and Figure 2E). Visual hallucinations were the most severe type of hallucinations across the course of the disease but were less severe than in *C9orf72* carriers (mean severity score at 0-4 years: 0.09 [95% CI, 0.01-0.17] points; at 4-8 years: 0.19 [95% CI, 0.12-0.23] points; at 8-12 years: 0.20 [95% CI, 0.09-0.29] points) (eFigure 2B in the Supplement).

#### MAPT

As with *GRN* carriers, anxiety and depression were preponderant in the early phases and less severe than in other gene variant groups, with anxiety steadily increasing during the course of the disease (mean severity score at 0-4 years: 0.38 [95% CI, 0.28-0.48] points; at 4-8 years: 0.58 [95% CI, 0.42-0.74] points; at 8-12 years: 0.97 [95% CI, 0.88-1.05] points), whereas depression increased only in the later stages (mean severity score at 4-8 years: 0.31 [95% CI, 0.17-0.46] points; at 8-12 years: 0.61 [95% CI, 0.47-0.76] points). Delusions, and particularly hallucinations, were significantly less frequent than in *C9orf72* and *GRN* carriers (mean severity score for hallucinations at 0-4 years: 0.02 [95% CI, 0.00-0.04] points in *MAPT* carriers vs 0.14 [95% CI, 0.06-0.21] points in *GRN* carriers and 0.41 [95% CI, 0.25-0.57] points in *C9orf72* carriers) (Table 2 and Figure 2F). In terms of the nature of hallucinations, visual hallucinations accounted for the majority of this symptom (mean severity score at 0-4 years: 0.02 [95% CI, 0.00-0.16] points; at 4-8 years: 0.05 [95% CI, 0.01-0.17] points; at 8-12 years: 0.06 [95% CI, 0.01-0.18] points) (eFigure 2C in the Supplement).

## Discussion

Behavioral and neuropsychiatric symptoms occur frequently over the course of many neurodegenerative disorders, but they are a core feature in FTD.^5,15^ A number of studies have focused on the progression of behavioral disturbances in the symptomatic phase of sporadic FTD and some in the presymptomatic phases of genetic FTD.^14,15,16,17,18,19,20,21,28,29,30,31,32^ However, to our knowledge, studies are lacking in the frequency and evolution of these symptoms during the symptomatic phases of monogenetic FTD. Furthermore, it has been unclear how different gene variations influence the nature and progression of these very important symptoms, which have relevant consequences for caregiver burden and quality of life.^33^

In the present study, we aimed to describe the relationship between the frequency and severity of behavioral and neuropsychiatric symptoms and disease progression in each of the main genetic variants associated with FTD, namely *C9orf72*, *GRN*, and *MAPT*. We observed in a large cohort of well-defined patients from the international GENFI study that frequencies and severity of behavioral symptoms may overlap and also differ significantly between gene variants.

Results suggest that behavioral symptoms, such as disinhibition, apathy, loss of empathy, perseverative and compulsive behavior, and hyperorality—the core symptoms of behavioral variant FTD—were expressed in all FTD pathogenic variations, with apathy being one of the most severe symptoms. Patients with the *MAPT* gene variant showed the highest frequency and severity of most core behavioral symptoms when compared with *C9orf72* and *GRN* carriers. However, alongside behavioral symptoms, results suggest that neuropsychiatric symptoms were also frequently reported in patients with genetic FTD. These manifestations, which are currently not defined as part of the FTD core symptoms, should be sought during evaluation.

Neuropsychiatric symptoms were particularly highly expressed in *C9orf72* carriers when compared with the other gene variations. Results suggest that anxiety predominated in the early phases of disease both in frequency and in severity, whereas hallucinations were more severe than any other symptom in the intermediate and late phases. In *GRN* carriers, depression and anxiety were predominant in both early and late phases of disease, whereas hallucinations and delusions were not common. Patients with the *MAPT* gene variants were highly affected by anxiety, whereas hallucinations and delusions were virtually absent.

Results suggest that hallucinations in different modalities were independently expressed in the different gene variants and should be sought because they could be highly suggestive of a particular genetic variation. In fact, *C9orf72* carriers were characterized by both auditory and visual hallucinations,^34^ with the former being more severe in the early-intermediate stages, whereas *GRN* carriers experienced mostly visual hallucinations. As reported previously, hallucinations were, however, not a distinctive feature of *MAPT* carriers.

The different behavioral and neuropsychiatric profiles largely reflected the discrete patterns of atrophy observed in each genetic variant.^32^ *C9orf72* and *GRN* carriers showed fewer differences in frequencies and trajectories of behavioral and neuropsychiatric symptoms between them when compared with *MAPT* carriers, possibly owing to their common underlying TAR DNA-binding protein 43 (TDP-43) proteinopathy, as opposed to tau pathology.^35^ Parallels between *C9orf72* and *GRN* carriers have also recently emerged regarding early cognitive symptoms,^31^ serum neurofilament light measures,^36^ and age at symptom onset and death,^37^ compared with *MAPT* carriers. This similarity could have crucial implications, because the direct comparison of symptoms among gene variant groups may be important in the consideration of basket-design clinical trials where, for example, patients with TDP-43 pathology arising from different gene variants (*C9orf72* and *GRN*) may be grouped together.^31^

These findings have important clinical implications. Knowledge of the pattern and prevalence of behavioral and neuropsychiatric symptoms over the course of the disease is particularly relevant for counseling patients and caregivers and for the evaluation of outcomes in FTD therapeutic trial designs. Behavioral disturbances evolve differently according to the particular gene variant, with relatively specific trajectories for each specific symptom. Knowing how symptoms evolve over the course of the disease could help the clinician and the caregiver in decisions regarding future management and therapeutic approaches.

### Limitations

We acknowledge that this study has some limitations. First, the number of assessments was more limited in advanced disease stages, possibly leading to some estimation errors. This limitation is in common with other observational studies and is almost inevitable owing to the high rate of institutionalization. Second, we cannot exclude possible effects of central nervous system–active drugs used differently in each genetic group. Furthermore, the present work did not cover all of the symptoms encountered during the course of the disease, as, for example, those reported in the Frontal Behavioral Inventory,^38,39^ such as restlessness, irritability, and aggression, as well as those related to aphasia and comprehension deficits. Nevertheless, we evaluated the core symptoms that define the criteria for behavioral variant FTD and that are virtually always encountered during the disease. Considering the wide variability of symptoms in patients with genetic FTD, even within individuals with the same gene variation in the same family, generalization of these results to single patients could be misleading. The current analysis does, however, represent one of the largest and best characterized studies in monogenic FTD to our knowledge. Further studies should assess the actual correspondence between these models and the observed symptoms during the natural history of the disease.

## Conclusions

In conclusion, the results of this cohort study suggest that behavioral and neuropsychiatric disturbances differ between the common FTD gene variations and have different trajectories through the course of the disease. This finding has crucial implications for counseling patients and caregivers and is very important for the design of disease-modifying treatment trials in genetic FTD.

## Notes

Supplement. **eMethods.** *GRN* and *MAPT* Pathogenic Variants Inclusion and Exclusion Criteria **eFigure 1.** Circos Plots for Behavioral and Neuropsychiatric Symptoms With Significant Pairwise Comparisons **eFigure 2.** Predicted Hallucinations’ (A-C) Severity According to Disease Duration in *C9orf72* Expansion Carriers, *GRN* and *MAPT* Carriers **eTable 1.** *GRN* Variants Included in the Study, Found in PubMed Search if Not Previously Reported in the “Alzheimer’s Disease & Frontotemporal Dementia Mutation Database” **eTable 2.** *MAPT* Variants Included in the Study, Found in PubMed Search if Not Previously Reported in the “Alzheimer’s Disease & Frontotemporal Dementia Mutation Database” **eTable 3.** Number of Evaluations in Each Genetic Group **eTable 4.** Interaction Terms of the Models **eReferences**
