# Error in Article Information

**DOI:** 10.1001/jamanetworkopen.2021.7664

**Published:** 2021-03-31

The Original Investigation titled “Progression of Behavioral Disturbances and Neuropsychiatric Symptoms in Patients With Genetic Frontotemporal Dementia,”^1^ published January 6, 2021, has been corrected to include the nonauthor collaborator names in a supplement.
