# Supplementary material for: Progression of Behavioral Disturbances and Neuropsychiatric Symptoms in Patients With Genetic Frontotemporal Dementia
Source: JAMA Netw Open. 2021 Jan 6;4(1):e2030194. doi: 10.1001/jamanetworkopen.2020.30194 (PMC7788468; doi:10.1001/jamanetworkopen.2020.30194)
Supplement: Supplement. — eMethods. GRN and MAPT Pathogenic Variants Inclusion and Exclusion Criteria eFigure 1. Circos Plots for Behavioral and Neuropsychiatric Symptoms With Significant Pairwise Comparisons eFigure 2. Predicted Hallucinations’ (A-C) Severity According to Disease Duration in C9orf72 Expansion Carriers, GRN and MAPT Carriers eTable 1. GRN Variants Included in the Study, Found in PubMed Search if Not Previously Reported in the “Alzheimer’s Disease & Frontotemporal Dementia Mutation Database” eTable 2. MAPT Variants Included in the Study, Found in PubMed Search if Not Previously Reported in the “Alzheimer’s Disease & Frontotemporal Dementia Mutation Database” eTable 3. Number of Evaluations in Each Genetic Group eTable 4. Interaction Terms of the Models eReferences [file jamanetwopen-e2030194-s001.pdf]

Supplementary Online Content

Benussi A, Premi E, Gazzina S, et al; Genetic FTD Initiative (GENFI). Progression of behavioral disturbances and neuropsychiatric symptoms in patients with genetic frontotemporal dementia. *JAMA Netw Open*. 2021;4(1):e2030194. doi:10.1001/jamanetworkopen.2020.30194

eMethods. *GRN* and *MAPT* Pathogenic Variants Inclusion and Exclusion Criteria

eFigure 1. Circos Plots for Behavioral and Neuropsychiatric Symptoms With Significant Pairwise Comparisons

eFigure 2. Predicted Hallucinations' (A-C) Severity According to Disease Duration in *C9orf72* Expansion Carriers, *GRN* and *MAPT* Carriers

eTable 1. *GRN* Variants Included in the Study, Found in PubMed Search if Not Previously Reported in the "Alzheimer's Disease & Frontotemporal Dementia Mutation Database"[1]

eTable 2. *MAPT* Variants Included in the Study, Found in PubMed Search if Not Previously Reported in the "Alzheimer's Disease & Frontotemporal Dementia Mutation Database"[1]

eTable 3. Number of Evaluations in Each Genetic Group

eTable 4. Interaction Terms of the Models

eReferences.

This supplementary material has been provided by the authors to give readers additional information about their work.

© 2021 Benussi A et al. *JAMA Network Open.*

eMethods. *GRN* and *MAPT* Pathogenic Variants Inclusion and Exclusion Criteria

GRN and MAPT pathogenic variants included in the study

GRN and *MAPT* variants were included if reported in the "Alzheimer's disease & Frontotemporal Dementia Mutation Database" (www.molgen.ua.ac.be/FTDmutations)[1] or published in peer-reviewed journals available in PubMed. The 29 *GRN* and 11 *MAPT* variants included in the study are shown with references in eTable 1 (*GRN*) and eTable 2 (*MAPT*) below.

Inclusion/exclusion criteria

For *GRN* and *MAPT,* variants causing haploinsufficiency, due to a frameshift mutation or insertion of a stop codon, were all included as likely pathogenic. The literature on *GRN* missense variants is less clear as to whether these are likely to be pathogenic or represent risk factors. We only included missense variants where there was evidence in the literature of *a)* low progranulin levels (in blood or CSF) similar to those causing haploinsufficiency (rather than intermediate levels as seen in some missense variants), or *b)* functional evidence of pathogenicity, and *c)* no contrary evidence that the variant was not pathogenic (i.e. the C139R variant has been shown to be associated with Alzheimer's disease pathology rather than TDP-43 inclusions as would be expected for *GRN* variants). For *C9orf72*, GGGGCC hexanucleotide repeat expansions were considered pathogenic only when >30 repeats were found in one of the two alleles.[2] *C9orf72* families with intermediate length expansions (24-30 repeats) were not included in the study.

© 2021 Benussi A et al. *JAMA Network Open.*

eFigure 1. Circos Plots for Behavioral and Neuropsychiatric Symptoms With Significant Pairwise Comparisons

Disinhibition.

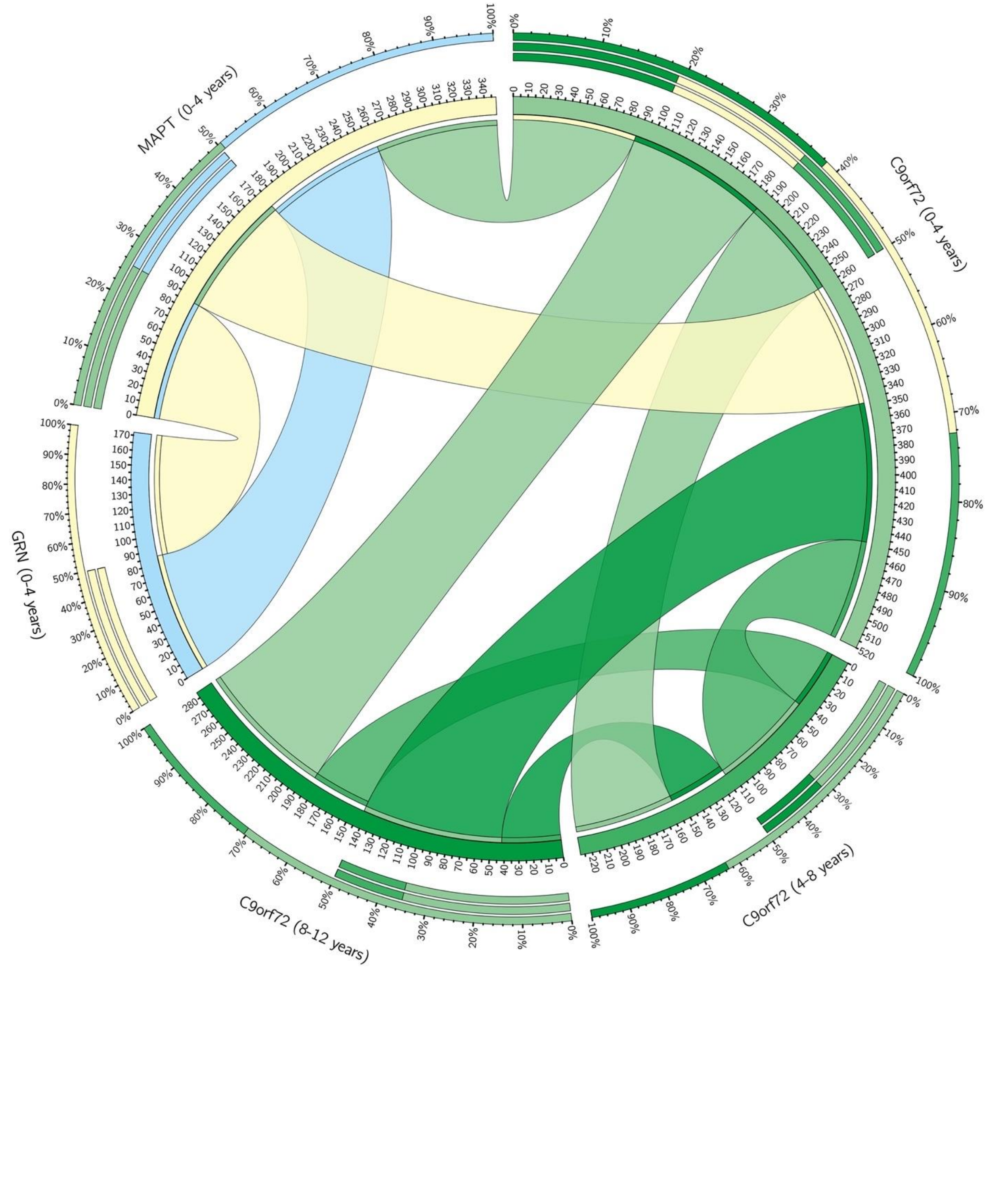

© 2021 Benussi A et al. *JAMA Network Open.*

Apathy.

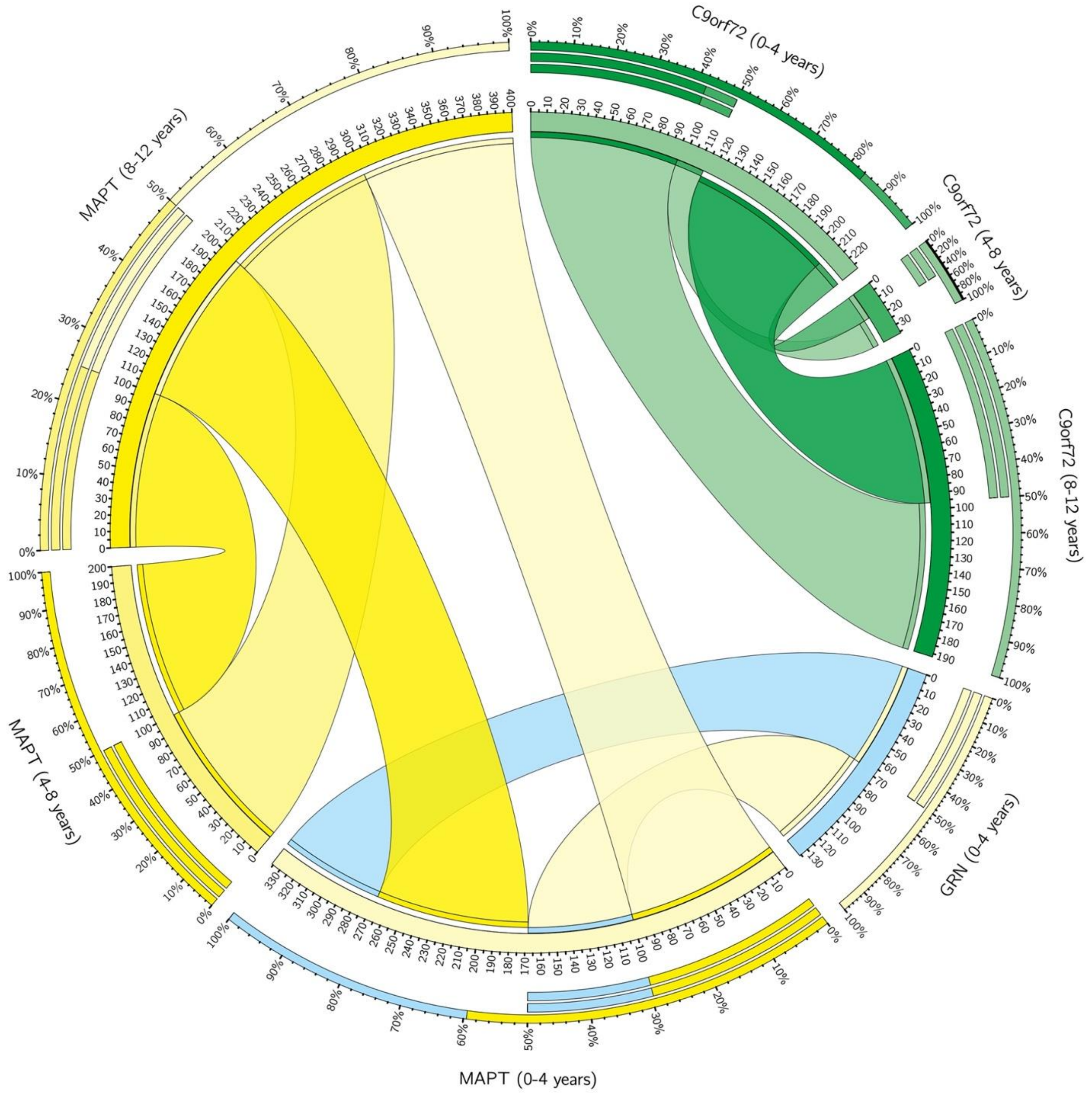

© 2021 Benussi A et al. *JAMA Network Open.*

Loss of empathy.

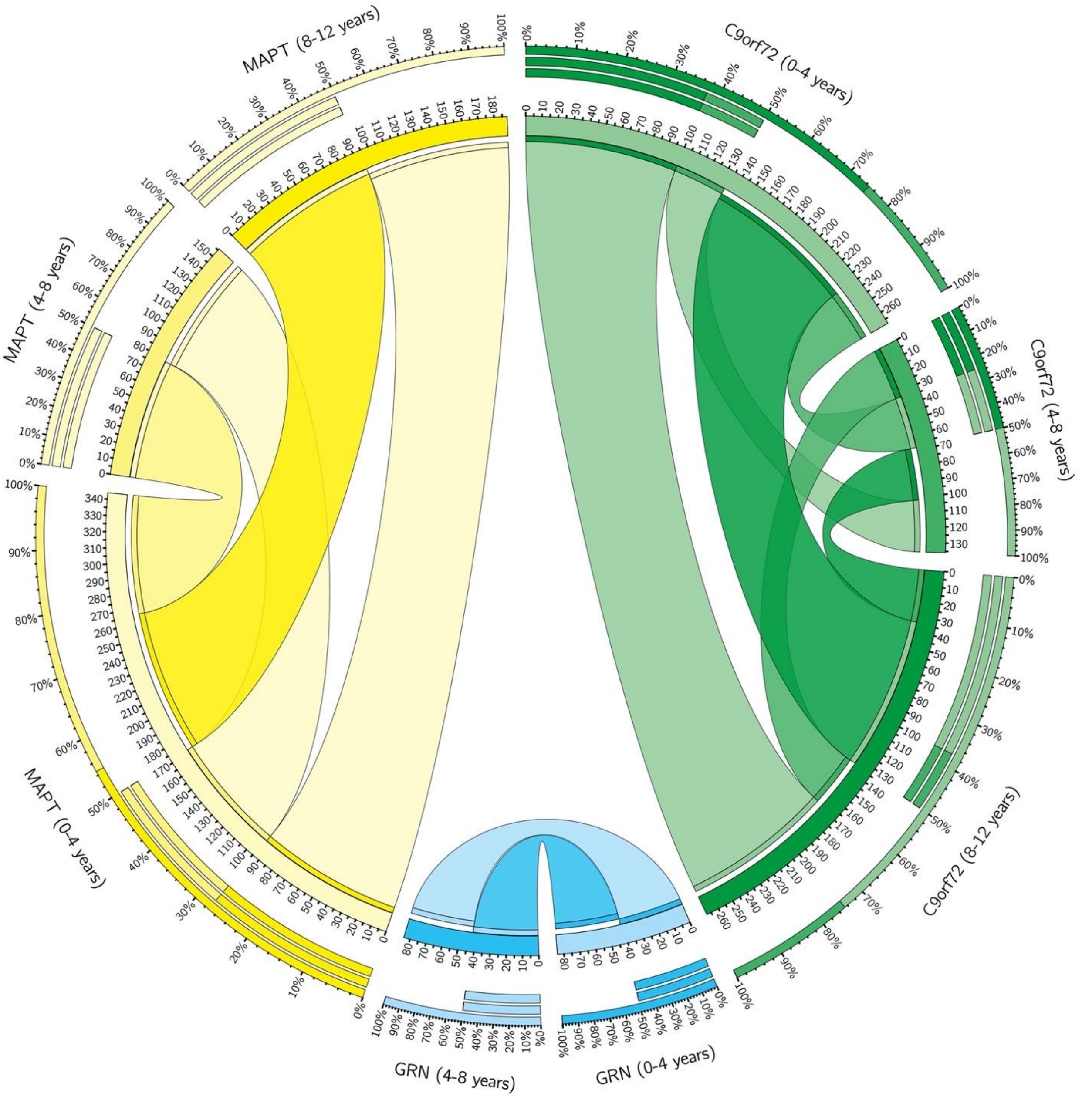

© 2021 Benussi A et al. *JAMA Network Open.*

Compulsive behavior.

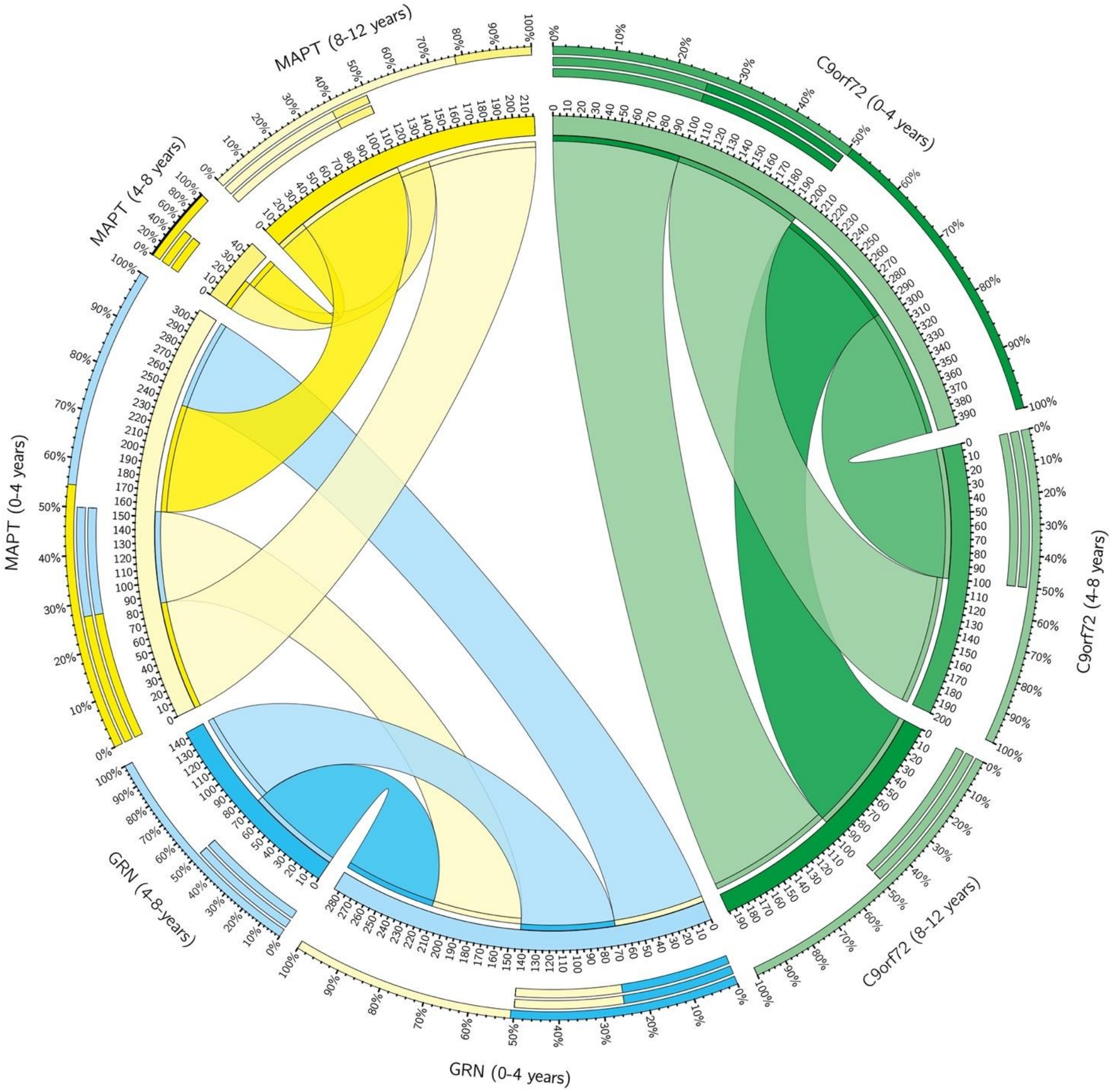

© 2021 Benussi A et al. *JAMA Network Open.*

Hyperorality.

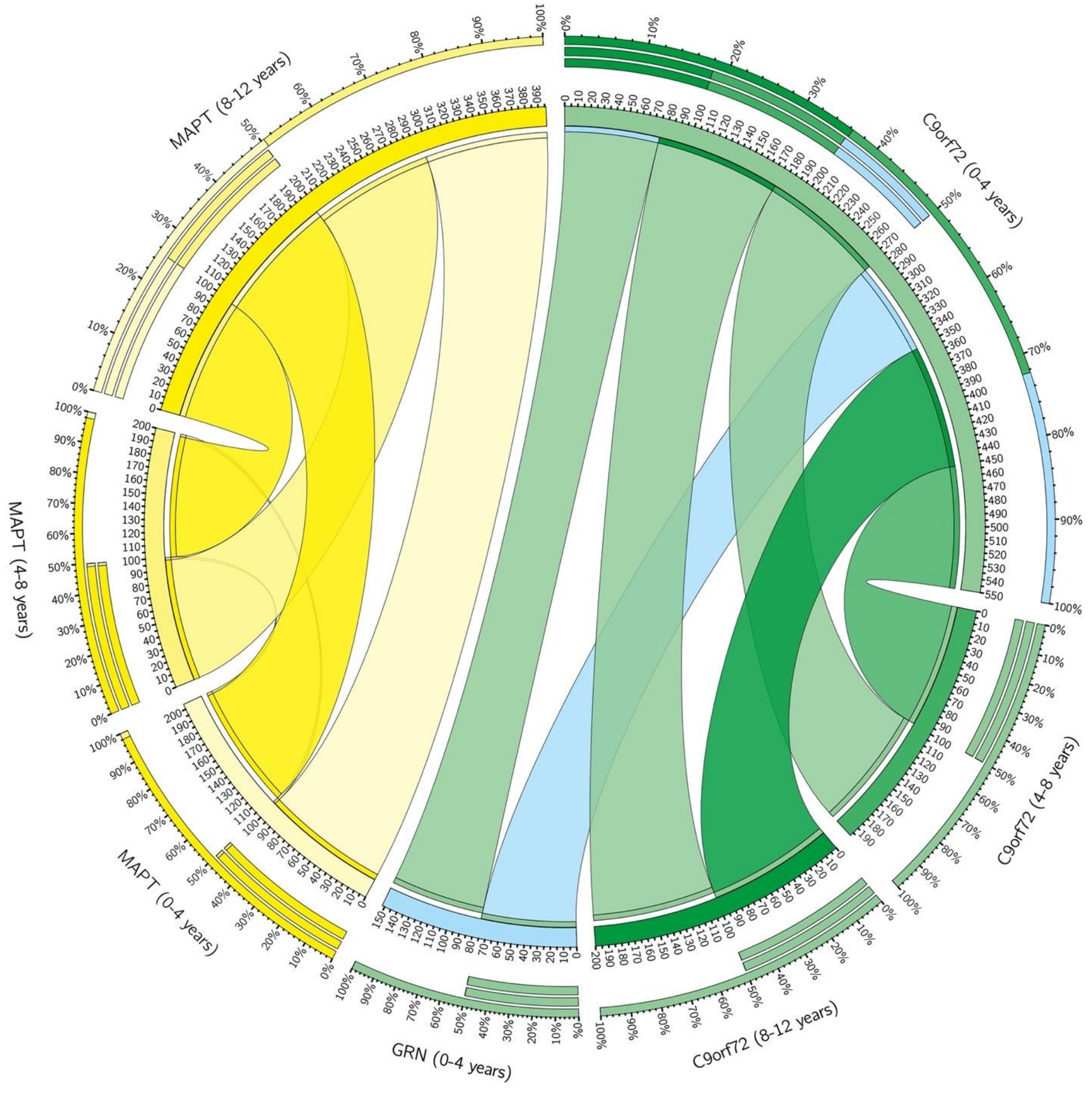

© 2021 Benussi A et al. *JAMA Network Open.*

Hallucinations.

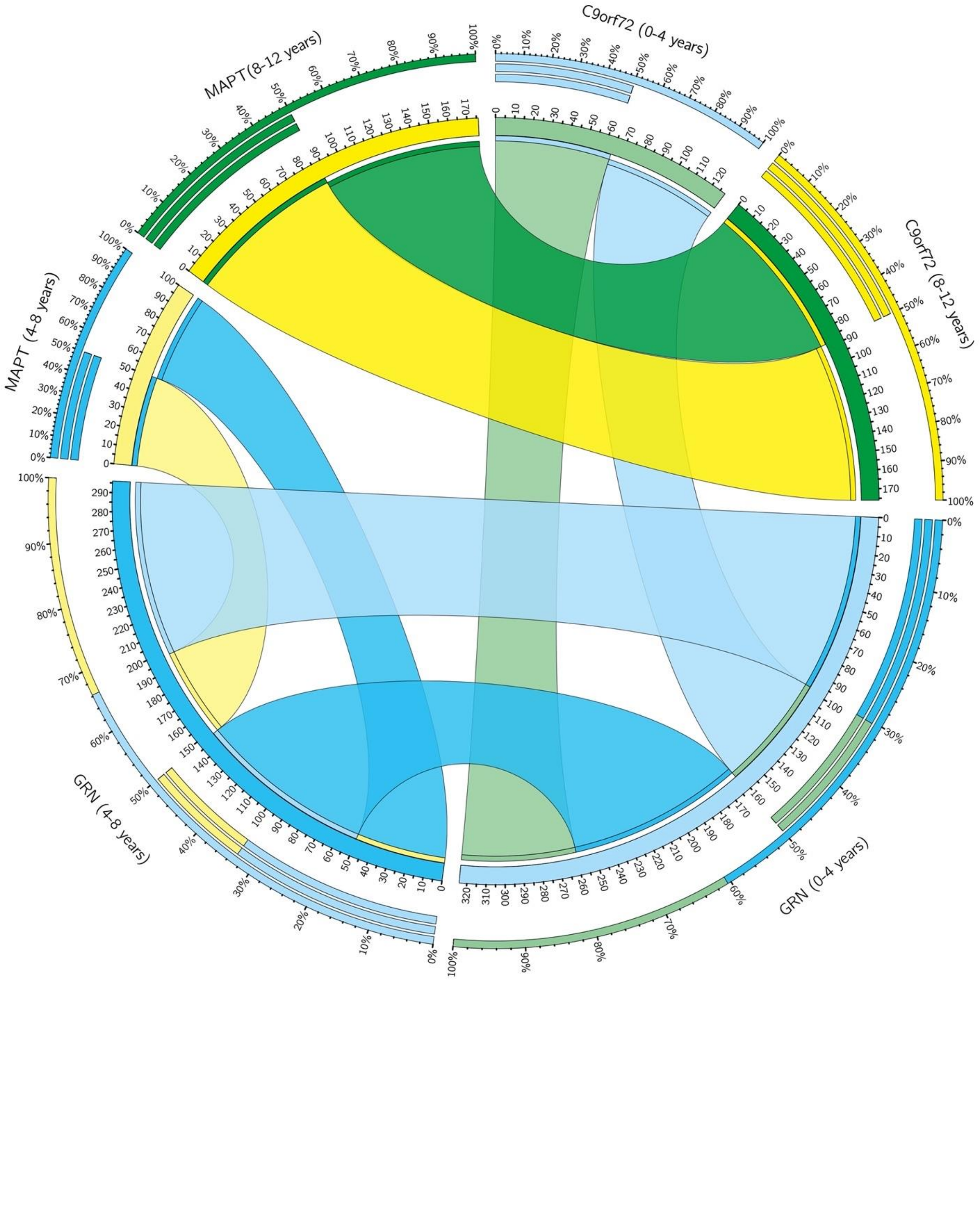

© 2021 Benussi A et al. *JAMA Network Open.*

Delusions.

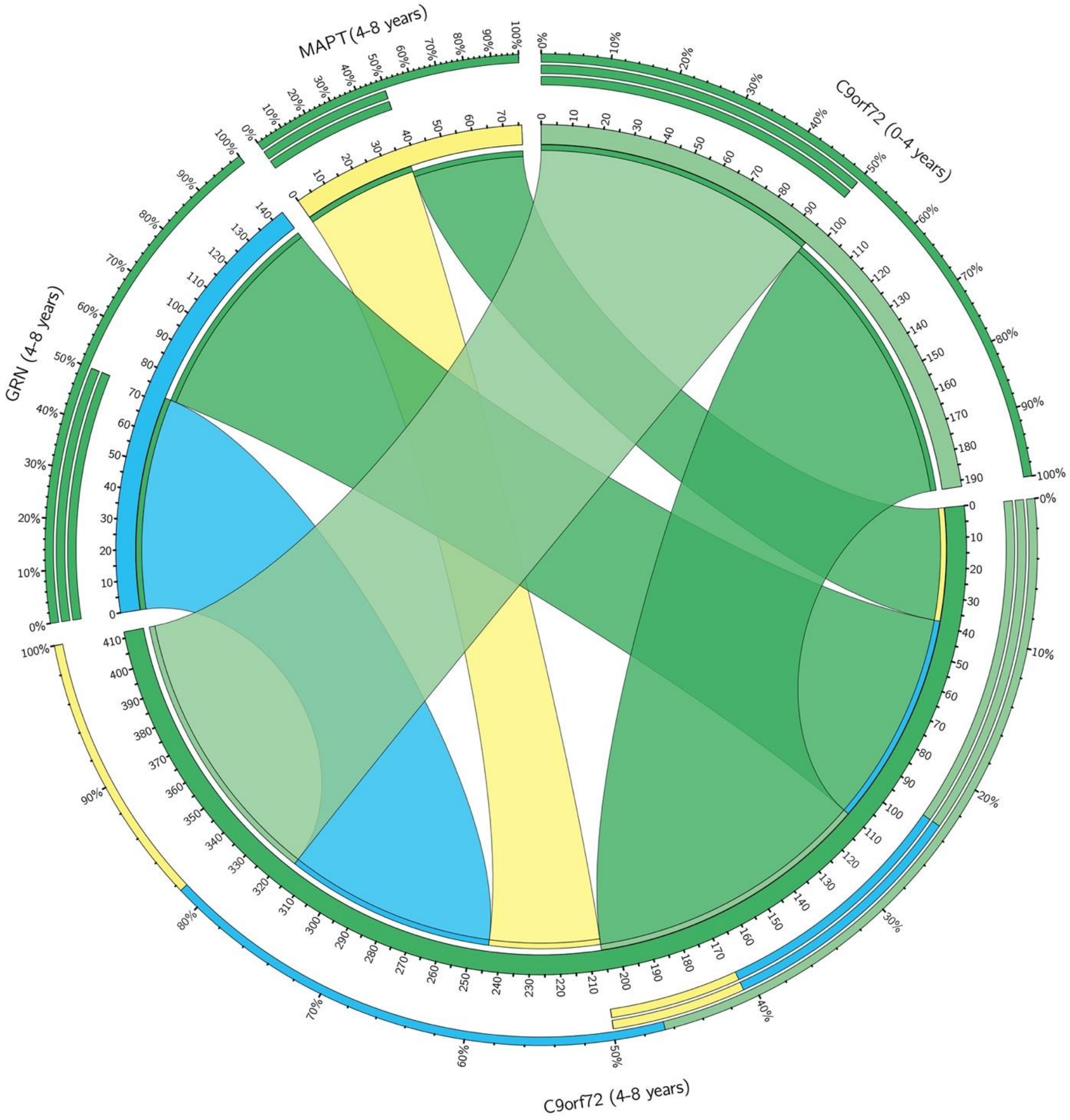

© 2021 Benussi A et al. *JAMA Network Open.*

Depression.

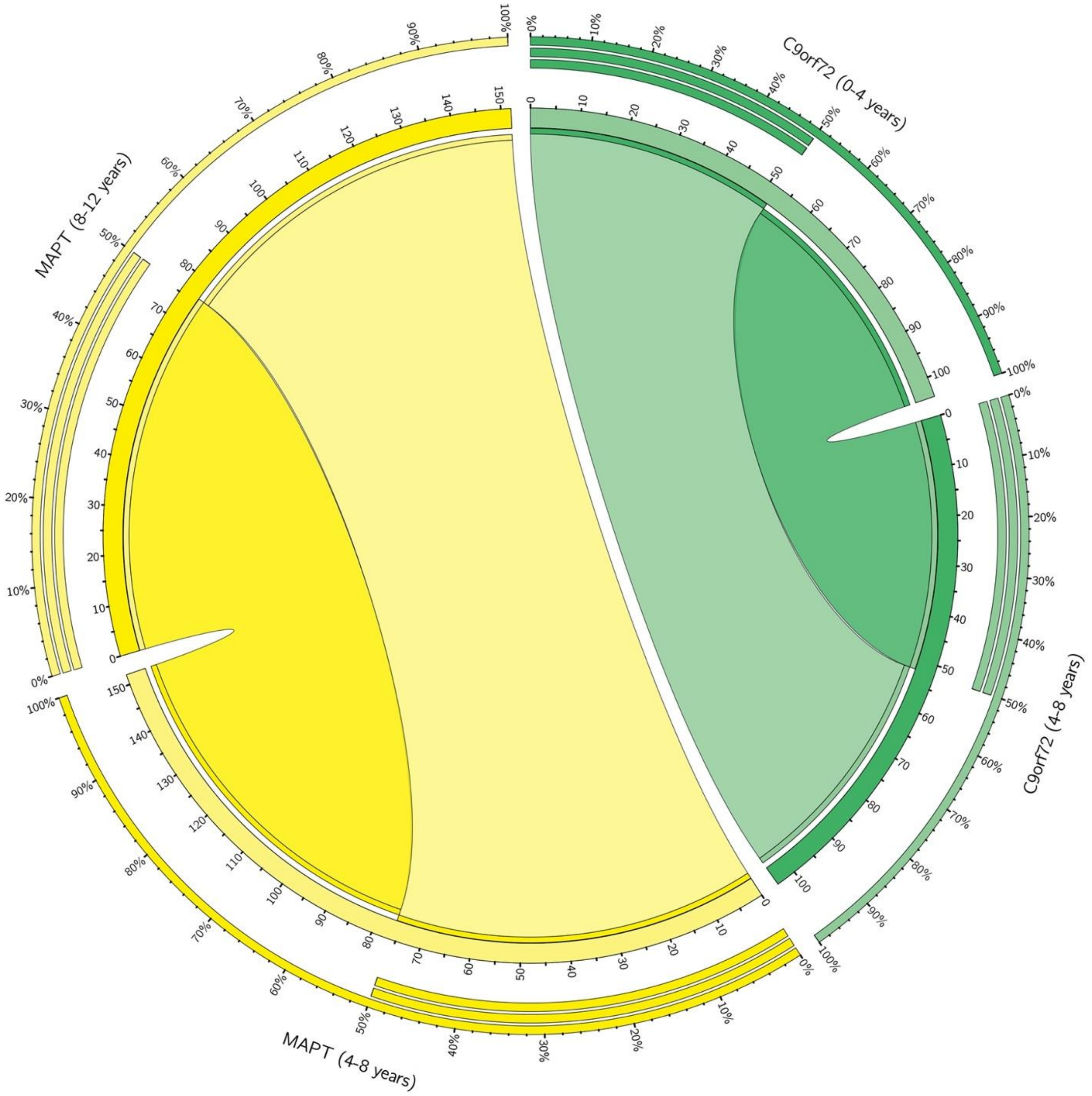

© 2021 Benussi A et al. *JAMA Network Open.*

Anxiety.

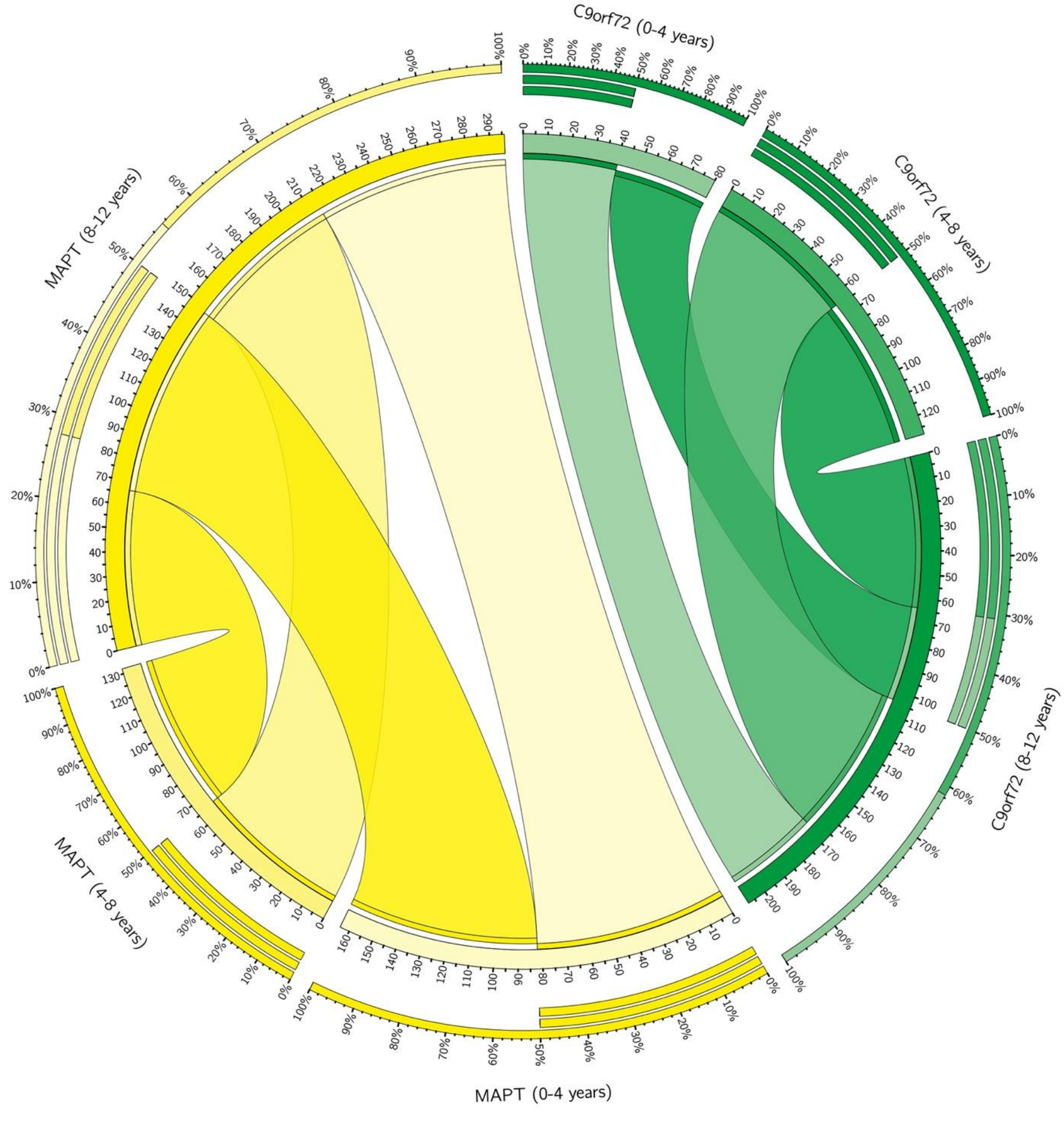

Ribbon sizes reflect strength of associations according to the formula: *p=(-n/2000)+0.05*
n stands for the digits in the inner circle.
Plots were created using the Circos visualization tool.[15]

© 2021 Benussi A et al. *JAMA Network Open.*

eFigure 2. Predicted Hallucinations' (A-C) Severity According to Disease Duration in *C9orf72* Expansion Carriers, *GRN* and *MAPT* Carriers

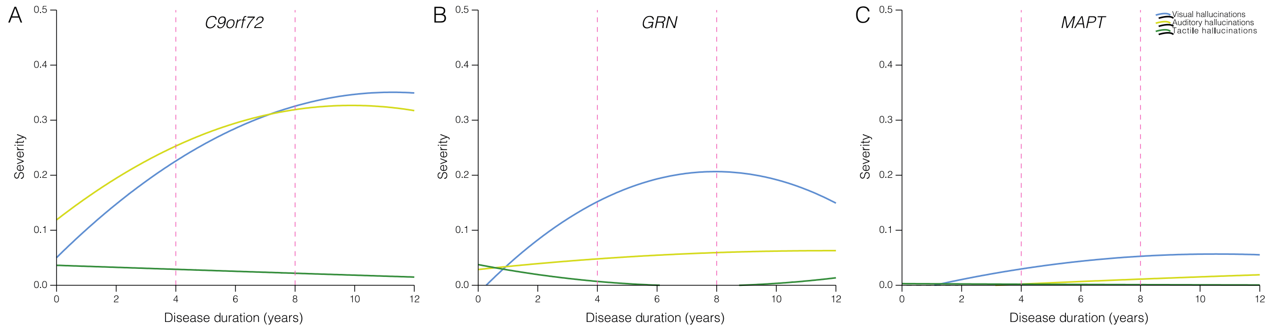

C9orf72 = chromosome 9 open reading frame 72; *GRN* = granulin; *MAPT* = microtubule-associated protein tau.

© 2021 Benussi A et al. *JAMA Network Open.*

eTable 1. *GRN* Variants Included in the Study, Found in PubMed Search if Not Previously Reported in the "Alzheimer's Disease & Frontotemporal Dementia Mutation Database".[1]

Variant Alias	Frequency	Reference
A350fs	1	3
C149fs	3	4
C253X	1	1,5
C31fs	3	1,5
C366fs	1	1,5
C416fs	1	6
C474fs	1	1,5
C482X	1	5
D254fs	1	5
delGRN[Tübingen]	1	5
G35fs	2	1,5
IVS7-1G>A	10	1,5
IVS7-2A>G	1	1,5
IVS7+6_9delTGAG	1	7–9
IVS8-1G>T	1	10
M1 (2T>C)	1	1,5
Q125X	1	1,5
Q130fs (388_391delCAGT)	3	1,5
Q249X	1	5
Q257fs	2	1,5
Q358X	1	1,5
R198fs	1	1,5
R418X	1	1,5
R493X	1	1,5

© 2021 Benussi A et al. *JAMA Network Open.*

S203fs	1	7,11
S78fs	1	5
S82fs	9	1,5
T272fs	25	1,5
W304fs (909delC)	1	1,5

eTable 2. *MAPT* Variants Included in the Study, Found in PubMed Search if Not Previously Reported in the "Alzheimer's Disease & Frontotemporal Dementia Mutation Database".[1]

Variant Alias	Frequency	Reference
G272V	3	1,5
IVS10+16	12	1,5
K257T	1	1,5
L266V	1	1,5
L284R	1	12
P301L	8	1,5
P301S	1	1,5
P397S	2	13
Q351R	2	14
R406W	7	1,5
V363I	1	1,5

© 2021 Benussi A et al. *JAMA Network Open.*

eTable 3. Number of Evaluations in Each Genetic Group

Evaluation	Total (n=400)	*C9orf72* (n=198)	*GRN* (n=121)	*MAPT* (n=81)
Baseline	232 (58.0%)	115 (58.1%)	78 (64.5%)	39 (48.1%)
Two evaluations	101 (25.3%)	53 (26.8%)	29 (24.0%)	19 (23.5%)
Three evaluations	35 (8.8%)	15 (7.6%)	10 (8.3%)	10 (12.3%)
Four evaluations	15 (3.8%)	7 (3.5%)	2 (1.7%)	6 (7.4%)
Five evaluations	8 (2.0%)	4 (2.0%)	1 (0.8%	3 (3.7%)
Six evaluations	7 (1.8%)	3 (1.5%)	1 (0.8%)	3 (3.7%)
Seven evaluations	3 (0.8%)	2 (1.0%)	0 (0.0%)	1 (0.0%)

© 2021 Benussi A et al. *JAMA Network Open.*

eTable 4. Interaction Terms of the Models

Outcome	Terms in the model	BIC
Disinhibition	Mutation, Duration, Mutation*Duration	1,019.26
Apathy	Mutation, Duration, Mutation*Duration	1,118.33
Loss of Empathy	Mutation, Duration, Mutation*Duration	1,119.54
Compulsive behavior	Mutation, Duration, Mutation*Duration	1,108.06
Hyperorality	Mutation, Duration, Mutation*Duration	1,103.10
Hallucinations	Mutation, Duration, Mutation*Duration	930.27
Delusions	Mutation, Duration, Mutation*Duration	761.89
Depression	Mutation, Duration, Mutation*Duration	834.46
Anxiety	Mutation, Duration, Mutation*Duration	899.24

The terms in each model of all possible 2-factor, 3-factor interaction terms along with second and third order disease duration terms were examined to reach a final model that fitted the data well for each marker.

BIC: Bayesian information criterion

© 2021 Benussi A et al. *JAMA Network Open.*

eReferences

1. Cruts M, Theuns J, Van Broeckhoven C. Locus-specific mutation databases for neurodegenerative brain diseases. *Hum Mutat*. 2012;33(9):1340-1344. http://onlinelibrary.wiley.com.atena-eco.unibs.it/doi/10.1002/humu.22117/pdf
2. Dejesus-Hernandez M, Mackenzie IR, Boeve BF, et al. Expanded GGGGCC Hexanucleotide Repeat in Noncoding Region of C9ORF72 Causes Chromosome 9p-Linked FTD and ALS. *Neuron*. Published online 2011:1-12. http://dx.doi.org/10.1016/j.neuron.2011.09.011
3. Rohrer JD, Beck J, Warren JD, et al. Corticobasal syndrome associated with a novel 1048_1049insG progranulin mutation. *J Neurol Neurosurg Psychiatry*. 2009;80(11):1297-1298. http://eutils.ncbi.nlm.nih.gov/entrez/eutils/elink.fcgi?dbfrom=pubmed&id=19864668&retmode=ref&cmd=prlinks
4. Calvi A, Cioffi SMG, Caffarra P, et al. The novel GRN g.1159_1160delTG mutation is associated with behavioral variant frontotemporal dementia. *J Alzheimers Dis*. 2015;44(1):277-282. http://eutils.ncbi.nlm.nih.gov/entrez/eutils/elink.fcgi?dbfrom=pubmed&id=25261445&retmode=ref&cmd=prlinks
5. Moore KM, Nicholas J, Grossman M, et al. Age at symptom onset and death and disease duration in genetic frontotemporal dementia: an international retrospective cohort study. *Lancet Neurol*. 2020;19(2):145-156. doi:10.1016/S1474-4422(19)30394-1
6. Chiang H-H, Forsell C, Lilius L, et al. Novel progranulin mutations with reduced serum-progranulin levels in frontotemporal lobar degeneration. *Eur J Hum Genet*. 2013;21(11):1260-1265. http://www.nature.com/doifinder/10.1038/ejhg.2013.37
7. Clot F, Rovelet-Lecrux A, Lamari F, et al. Partial deletions of the GRN gene are a cause of frontotemporal lobar degeneration. *Neurogenetics*. Published online January 28, 2014. doi:10.1007/s10048-014-0389-x
8. Bit-Ivan EN, Suh E, Shim H-S, et al. A Novel GRN Mutation (GRN c.708+6_+9delTGAG) in Frontotemporal Lobar Degeneration With TDP-43–Positive Inclusions. *J Neuropathol Exp Neurol*. 2014;73(5):467-473. http://content.wkhealth.com/linkback/openurl?sid=WKPTLP:landingpage&an=00005072-201405000-00009
9. Guven G, Lohmann E, Bras J, et al. Mutation frequency of the major frontotemporal dementia genes, MAPT, GRN and C9ORF72 in a Turkish cohort of dementia patients. *PLoS One*. 2016;11(9):1-16. doi:10.1371/journal.pone.0162592
10. Antonell A, Gil S, Sanchez-Valle R, et al. Serum progranulin levels in patients with frontotemporal lobar degeneration and Alzheimer's disease: detection of GRN mutations in a Spanish cohort. *J Alzheimers Dis*. 2012;31(3):581-591.
© 2021 Benussi A et al. *JAMA Network Open.*

http://eutils.ncbi.nlm.nih.gov/entrez/eutils/elink.fcgi?dbfrom=pubmed&id=22647257&retmode=ref&cmd=prlinks

11. Schofield EC, Halliday GM, Kwok J, Loy C, Double KL, Hodges JR. Low serum progranulin predicts the presence of mutations: a prospective study. *J Alzheimers Dis*. 2010;22(3):981-984. http://eutils.ncbi.nlm.nih.gov/entrez/eutils/elink.fcgi?dbfrom=pubmed&id=20858962&retmode=ref&cmd=prlinks

12. Rohrer JD, Paviour D, Vandrovcova J, Hodges J, de Silva R, Rossor MN. Novel L284R MAPT Mutation in a Family with an Autosomal Dominant Progressive Supranuclear Palsy Syndrome. *Neurodegener Dis*. 2011;8(3):149-152. http://www.karger.com/doi/10.1159/000319454

13. Borrego-Écija S, Antonell A, Puig-Butillé JA, et al. Novel P397S MAPT variant associated with late onset and slow progressive frontotemporal dementia. *Ann Clin Transl Neurol*. 2019;6(8):1559-1565. doi:10.1002/acn3.50844

14. Liang Y, Gordon E, Rohrer J, et al. A cognitive chameleon: Lessons from a novel MAPTmutation case. *Neurocase*. 2013;20(6):684-694. http://www.tandfonline.com/doi/abs/10.1080/13554794.2013.826697

15. Krzywinski M, Schein J, Birol I, et al. Circos: An information aesthetic for comparative genomics. *Genome Res*. 2009;19(9):1639-1645. doi:10.1101/gr.092759.109

© 2021 Benussi A et al. *JAMA Network Open.*
